# IGF1R Induces ROS Formation in Mitochondria via MICU1 Leads to the Enzalutamide Resistance in Prostate Cancer

**DOI:** 10.1111/jcmm.71415

**Published:** 2026-10-27

**Authors:** Hanlin Li, Yutong Chen, Yupeng Zhou, Yang Li, Yukui Gao, Xuanrong Chen, Qianwang Ma, Shimiao Zhu, Simeng Wen

**Affiliations:** ^1^ Department of Urology The Second Affiliated Hospital Zhejiang University School of Medicine Hangzhou China; ^2^ Tianjin Institute of Urology The Second Hospital of Tianjin Medical University Tianjin China

**Keywords:** Ca^2+^, IGF1R, metabolism, prostate cancer, ROS

## Abstract

After undergoing ADT treatment, drug resistance has emerged as a major hindrance in the treatment of prostate cancer, with enzalutamide resistance being the most notable. Our objective was to identify a new drug‐resistance mechanism present in post‐ADT‐treatment patients. Utilizing proteomic sequencing, we discovered abnormal increases in IGF1R levels. Subsequent sequencing and bioinformatic analyses showed that IGF1R could cause abnormal changes in the tricarboxylic acid cycle (TCA cycle) and oxidative phosphorylation. This phenomenon may be due to IGF1R's influence on the distribution of mitochondrial Ca^2+^ and the production of reactive oxygen species (ROS). Our CHIP and dual‐luciferase experiments confirmed the regulatory relationship between IGF1R and MICU1. The fluorescent results of Ca^2+^ and ROS also confirmed that IGF1R could regulate mitochondrial Ca^2+^ distribution and ROS production. Our team validated the relationship between IGF1R and drug resistance through cell phenotype experiments, and explored its potential as a therapeutic target through a fa‐ci curve. Finally, we verified the effectiveness of the mechanisms we found through a xenograft assay. Our study confirms the potential for IGF1R as a clinical therapeutic target.

**Abbreviations**
ADT: androgen‐deprivation therapy
C4‐2R: C4‐2 enzalutamide‐resistant
CHIP: chromatin immunoprecipitation
CI: combination index
CRPC: castration‐resistant prostate cancer
EMT: epithelial‐mesenchymal transition
FBS: fetal bovine serum
FISH: fluorescence in situ hybridization
LNR: LN‐CaP enzalutamide‐resistant
ROS: reactive oxygen species
TCA: tricarboxylic acid

## 1 Introduction

The rise in affluence has made cancer a significant threat to human health, with prostate cancer being a leading cause of death among men [1, 2]. While considerable progress has been made in its treatment, androgen‐deprivation therapy (ADT) remains a major therapeutic approach for advanced prostate cancer [3]. However, patients often develop resistance to ADT over time. Enzalutamide, a second‐generation antiandrogen drug, is widely used to treat castration‐resistant prostate cancer (CRPC), yet most patients will eventually experience resistance after an initial period of sensitivity [4]. Understanding the mechanisms underlying enzalutamide resistance is essential for identifying new therapeutic targets for advanced prostate cancer.

The IGF1R is a key phosphorylated receptor involved in cellular signalling and metabolism [5]. Located on the cell membrane, IGF1R serves as a classical upstream regulator in numerous phosphorylation signalling pathways [6]. MICU1, situated in the inner mitochondrial membrane, regulates the expression of the MCU [7] and plays a crucial role in maintaining mitochondrial homeostasis [8]. Given the link between mitochondrial calcium levels and reactive oxygen species (ROS) production, however, research examining the relationship between MICU1, ROS and ADT resistance is limited [9]. Further investigation into MICU1 in the context of ADT resistance could provide valuable insights into the progression of advanced prostate cancer.

## 2 Methods and Materials

### 2.1 Cell Culture and Constructs

To generate Enzalutamide‐resistant LNCaP and C4‐2 cells, these cell lines were subjected to continuous selection in the presence of 1 μM or 10 μM Enzalutamide (MedChemExpress) for at least 3 months. Mycoplasma‐free LNCaP, C4‐2, LNR and C4‐2R cells were cultured in RPMI 1640 medium supplemented with 10% fetal bovine serum (FBS), whereas COS7 cells were cultured in DMEM. All cell lines, except for LNR and C4‐2R, were obtained from ATCC. To maintain the drug‐resistant phenotypes, 20 μM Enzalutamide was added to the culture medium of C4‐2R cells during routine cultivation. For LNR cells, 10 μM Enzalutamide was included in the medium.

### 2.2 Proteomics, Phosphorylated Proteomics, Metabolomics and Whole Transcriptome Sequencing

Sequencing for this project was performed by Applied Protein Technology Company. Bioinformatics analysis was conducted using R Studio software. GeneCards [10] was utilized to identify potential transcription factors, while JASPAR [11] was used for predicting transcription factor binding sites. Non‐paired *t*‐tests were used to assess statistical differences between groups, with a significance threshold of *p* < 0.05.

### 2.3 RNA Extraction and First Strand cDNA Synthesis

Total RNA was extracted from cultured cells using Trizol reagent (ThermoFisher Scientific). For cells in a T25 flask, 1 mL of Trizol was used for RNA extraction. The extracted RNA was immediately reverse‐transcribed into cDNA using the RevertAid First Strand cDNA Synthesis Kit (ThermoFisher Scientific).

### 2.4 Primers, SiRNAs, Antibodies and Plasmids

The primers used in this study are as follows: ITGAE (F: AAATCCTTTGCCATCCTGTAGAG; R: CCGGACCAGCACTTGAATG), ACPP (F: CAGACGTTGACCGGACTTTGA; R: GCCAGAGTAGGATAGGATTCCA), NOS3 (F: TGATGGCGAAGCGAGTGAAG; R: ACTCATCCATACACAGGACCC), UTRN (F: TGAAGGATGTCATGTCGGACC; R: GTTGAGGACGTTGACTTGGCT), RELL2 (F: TCATGGGCCTGGTAGGCTT; R: TGTGTCCTCATTCATGTCATCG), MICU1 (F: AGAGCATCATTCGCTCCCAAA; R: GCAGTTTACGCTGAAATTCGAG), PRO‐MICU1 (F: CGGCTGCTCTGAATGTGTTA; R: TAGGAGGCAGAGGTTGCAGT), IGF1R (F: AGGATATTGGGCTTTACAACCTG; R: GAGGTAACAGAGGTCAGCATTTT), GAPDH (F: GGAGCGAGATCCCTCCAAAAT; R: GGCTGTTGTCATACTTCTCATGG).

All the siRNAs, antibodies and plasmids are shown in the Tables S1 and S2.

### 2.5 Reagent

Reagents used in this study but not mentioned elsewhere include: FastStart Universal SYBR Green Master (Roche); X‐tremeGENE siRNA transfection reagent (Roche); X‐tremeGENE HP DNA transfection reagent (Roche); DMSO (cell culture grade, Solarbio); DMSO (Solarbio); SDS‐PAGE Gel Kit (Solarbio); AKTi‐1/2 (Selleck).

### 2.6 Cell Proliferation and Migration Assays

Cell proliferation was assessed using the MTT assay. The combination of two drugs was evaluated by calculating the combination index (CI) following the manufacturer's guidelines (CompuSyn) [12]. For the cell migration assay, 8 × 10^3^ cells were seeded in the upper chamber with RPMI 1640, and 450 μL RPMI 1640 containing 20% FBS was added to the lower chamber. Cells were fixed with paraformaldehyde and stained with crystal violet. IC50 values and relative drug concentrations are provided in Table S3.

### 2.7 Fluorescence In Situ Hybridization (FISH)

Cells were fixed with paraformaldehyde, followed by treatment with 0.25% Triton X‐100. Blocking was performed using 5% BSA for 30 min. After overnight incubation with the primary antibody, cells were incubated with a fluorescent secondary antibody for 1 h at 37°C.

### 2.8 Chromatin Immunoprecipitation (CHIP)

ChIP assays were performed using the EZ‐Magna ChIP A/G kit (Millipore). Briefly, cells were harvested and fixed, and the nuclei were isolated and sonicated. The DNA was purified and immunoprecipitated. The results were analyzed by PCR.

### 2.9 Dual‐Luciferase Assay

Dual‐luciferase reporter assays were performed on transfected cells using the Dual‐Luciferase Reporter Assay System (Promega). Following cell lysis, the supernatant was collected after centrifugation. Both luciferase substrates were used, and fluorescence was measured using a GloMax 20/20 Luminometer.

### 2.10 Calcium Staining Assay

Calcium ion levels in cells were detected using Rhod‐3 and Fluo‐4 (ThermoFisher Scientific) according to the manufacturer's instructions. Staining was visualized using confocal microscopy.

### 2.11 ROS Detection

Intracellular ROS were measured using the ROS assay kit (Solarbio). For adherent cells, the culture medium was removed, and DCFH‐DA was added. Cells were incubated at 37°C for 20 min, and fluorescence was observed under a confocal microscope.

### 2.12 Xenograft Assay

Five‐week‐old BALB/c nude mice were used for the xenograft assay. Ten mice were randomly divided into two groups without blinding: a treatment group and a control group. The treatment group received intratumoural injections of Linsitinib (Selleck, OSI‐906), while the control group received DMSO. Tumour diameters were measured every 3 days. After 3 weeks, mice were euthanized, and tumour tissues were excised. All animal experiments were performed according to the guidelines of the Laboratory Animal Care Evaluation and Identification Association. Tumour volume was calculated using the formula: V = (π/6) × (longest diameter) × (shortest diameter)^2^.

### 2.13 Immunohistochemistry

Tissue sections were dried in an oven at 65°C for 45 min. Antigen retrieval was performed using sodium citrate, followed by 15 min of incubation with 3% H_2_O_2_ at 37°C. Primary antibodies were diluted in PBS and applied to the sections. After overnight incubation at 4°C, sections were incubated with secondary antibodies for 15 min at 37°C. Colour development was achieved using DAB, and nuclei were counterstained with haematoxylin. The sections were dehydrated and mounted.

### 2.14 Statistical Analyses

Data generation and analysis were performed using GraphPad Prism v9.0. Unpaired *t*‐tests were used to determine statistical significance between two datasets, unless otherwise specified. Bar values represent the mean ± standard deviation or standard error of the mean. All datasets were normally distributed. Abbreviations used in the figures denote statistical significance: ns (not significant), *p* ≤ 0.05, *p* ≤ 0.01, *p* ≤ 0.001, **p ≤ 0.0001.

## 3 Results

### 3.1 IGF1R Is Upregulated in Enzalutamide‐Resistance PCa

To better understand the mechanisms underlying enzalutamide resistance in prostate cancer, we established two enzalutamide‐resistant cell lines: C4‐2R (C4‐2 enzalutamide‐resistant) and LNR (LN‐CaP enzalutamide‐resistant). Proteomic sequencing was then performed on both C4‐2 and C4‐2R cell lines (Figure 1A,B). The sequencing results were categorized based on subcellular localization using UniProt annotation (Figure 1C) [13]. Our goal was to identify potential therapeutic targets, so we specifically focused on proteins that may be expressed on the cell membrane. From the proteomic data, we compiled a list of the 10 most differentially expressed cell membrane proteins (Figure 1D). Primers were designed for these differentially expressed proteins, and quantitative real‐time polymerase chain reaction (qPCR) was performed to verify their RNA expression levels. Based on these results, we selected IGF1R for further investigation (Figure 1E). Western blot analysis confirmed that IGF1R protein levels were significantly elevated in both C4‐2R and LNR cell lines (Figure 1F). To explore the relationship between IGF1R expression and enzalutamide resistance, we conducted IC50 experiments, referencing data from the Selleck company website for preliminary validation. First, the IC50 of enzalutamide‐sensitive C4‐2 cells was measured (Figure 1G), followed by testing in C4‐2R cells and in C4‐2R cells with IGF1R knockdown (Figure 1H,I). Similar experiments were performed on LNCaP cells and an additional cell line generated in our laboratory to further validate the results (Figure S1A–C).

**FIGURE 1:**
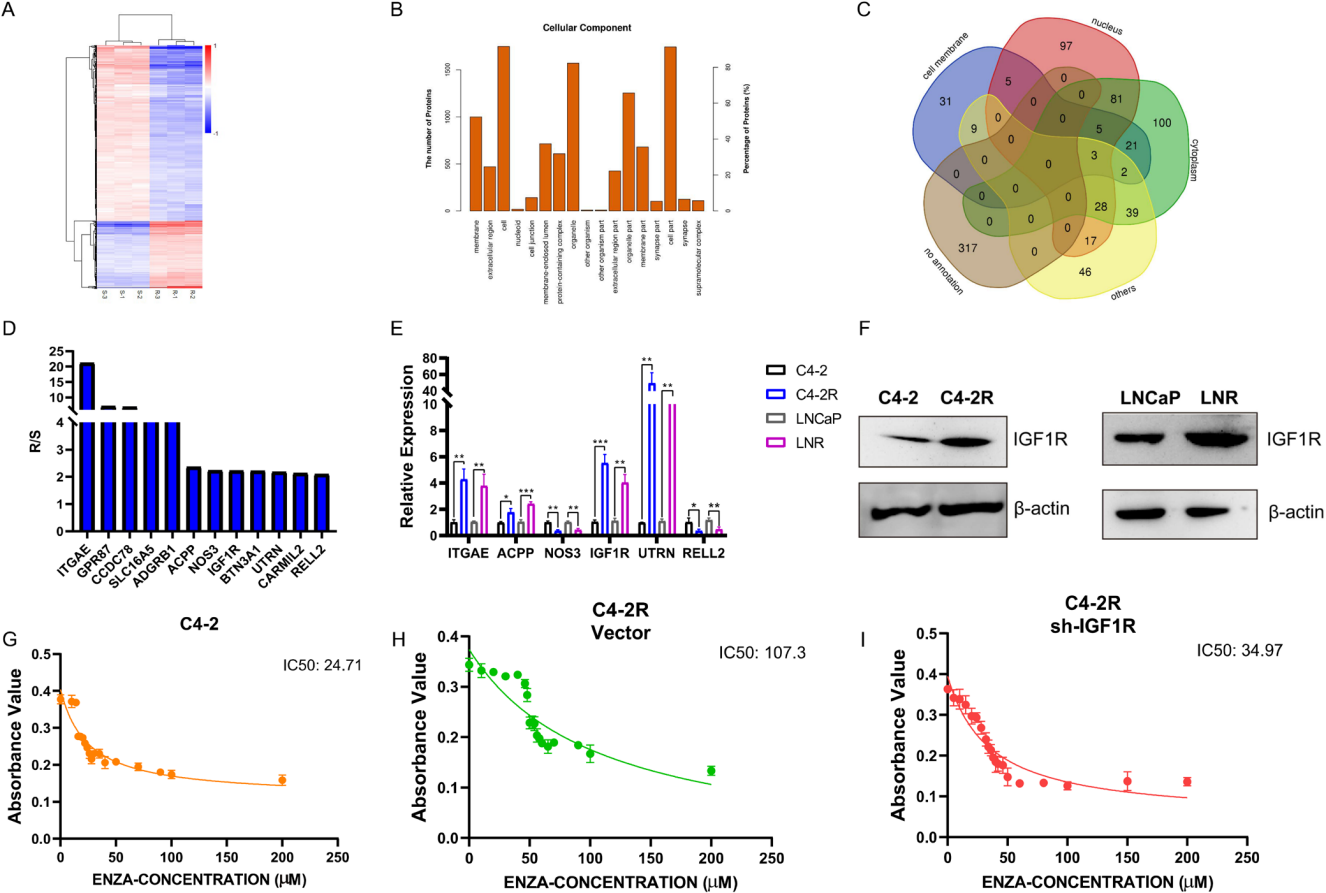
IGF1R is highly expressed in enzalutamide‐resistant cell lines. (A) A heatmap displaying the results of proteomic sequencing in C4‐2 and C4‐2R cells. (B) Classification of differential proteins according to cellular component. (C) Reclassification of differential proteins based on human protein annotations from the UniProt database, followed by the generation of a Venn diagram based on the obtained results. (D) A histogram illustrating the differential proteins classified into the cell membrane group in panel C. (E) Validation of the results shown in panel D using quantitative real‐time polymerase chain reaction (qPCR) across four cell lines. (*p < 0.05, **p < 0.01, ***p < 0.001). (F) Western blot experiments revealing IGF1R expression in various cell lines. Elevated expression of IGF1R was observed in C4‐2R and LNR cell lines. (G) IC50 results for the C4‐2 cell line. The X‐axis represents different enzalutamide concentrations, and the Y‐axis represents the absorbance of C4‐2 cells in the MTT assay at the corresponding enzalutamide concentrations. (H) IC50 results for the C4‐2R cell line. The X‐axis represents different enzalutamide concentrations, and the Y‐axis represents the absorbance of C4‐2R cells in the MTT assay at the corresponding enzalutamide concentrations. Panel I contrasts with this by showing results from C4‐2R cells transfected with a negative control plasmid. (I) IC50 results for C4‐2R cells with low IGF1R expression. The X‐axis represents different enzalutamide concentrations, and the Y‐axis represents the absorbance of sh‐IGF1R‐C4‐2R cells in the MTT assay at the corresponding enzalutamide concentrations.

### 3.2 IGF1R Regulates the TGF‐β Signalling Pathway Through Reactive Oxygen Species (ROS) Modulation

To investigate the role of IGF1R in cellular resistance to enzalutamide, we performed phosphoproteomic sequencing on C4‐2R cells with IGF1R knockdown (Figure 2A). Surprisingly, we observed a reduction in the phosphorylation levels of key proteins in the TGF‐β signalling pathway upon IGF1R knockdown (Figure 2B). We then conducted transcriptomic sequencing (Figure 2C), and through Gene Set Enrichment Analysis, we identified significant alterations in oxidative phosphorylation and hypoxia‐related pathways (Figure 2D,E) [14]. These pathways, while broadly implicated in various cellular changes, may relate to the modulation of ROS in the TGF‐β pathway [15]. To explore whether the changes in oxidative phosphorylation and hypoxia pathways lead to shifts in ROS levels, we conducted KEGG pathway analysis of the transcriptomic data, revealing notable alterations in the calcium signalling pathway, a crucial component of our proposed mechanism (Figure 2F). Based on these findings, we hypothesize that IGF1R may regulate the TGF‐β signalling pathway through its effects on cellular ROS levels. To further validate this hypothesis, we employed targeted metabolomics. Additionally, to strengthen our results, we overexpressed MICU1, a key protein involved in mitochondrial calcium regulation, in IGF1R knockdown cells, as suggested by the KEGG analysis. Our results showed that while IGF1R knockdown increased tricarboxylic acid cycle products, the NAD+/NADH ratio decreased, indicating reduced reducing equivalents available for downstream reactions (Figure 2G,H, Figure S2F). Based on these findings, we propose that IGF1R regulates the TGF‐β signalling pathway by modulating mitochondrial ROS production (Figure 2I).

**FIGURE 2:**
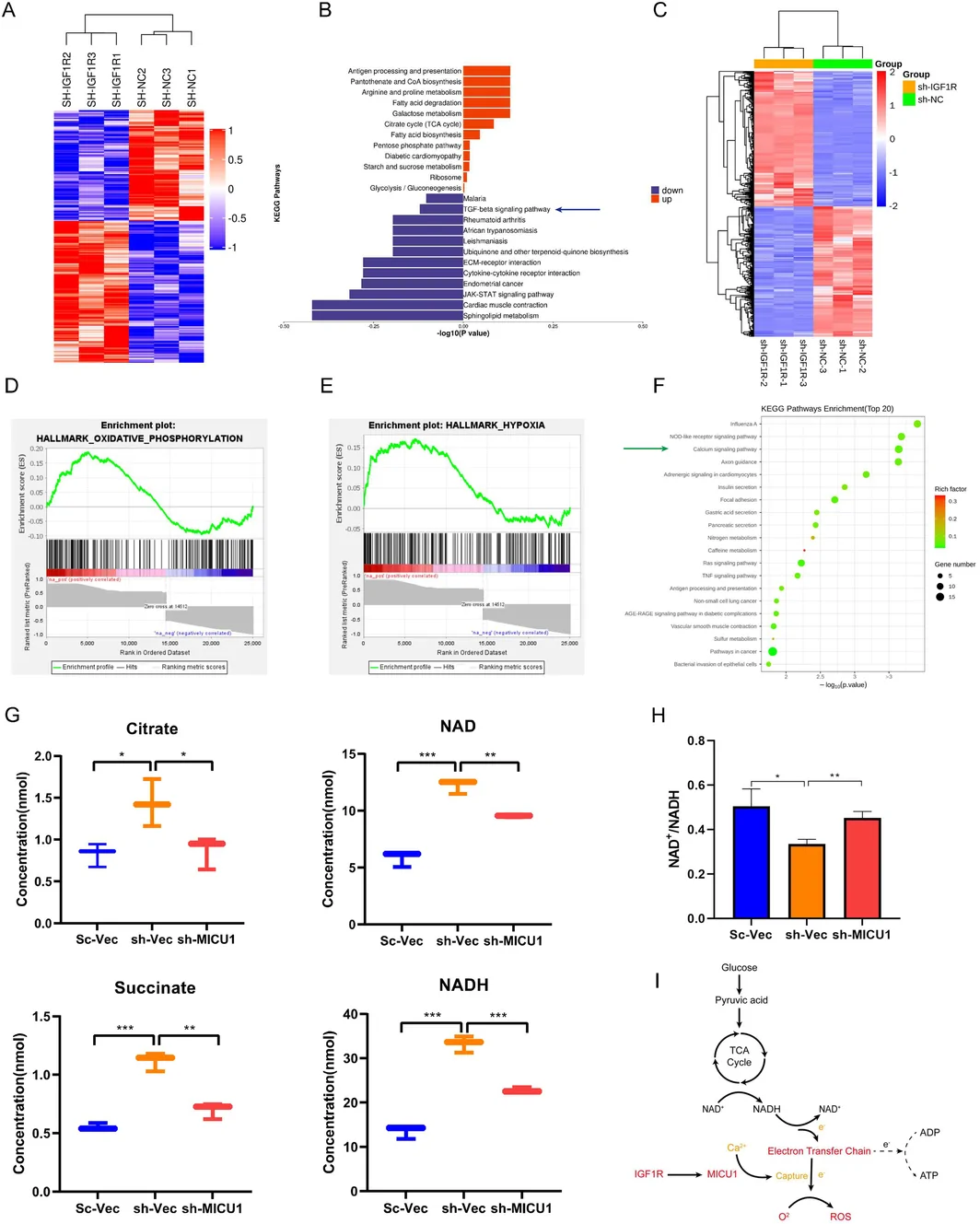
Knocking down IGF1R leads to increased expression of oxidative phosphorylation‐related proteins. (A) A heatmap displaying the results of phosphoproteomic sequencing in C4‐2R cells, comparing low IGF1R expression with the negative control. (B) KEGG enrichment analysis of phosphoproteomic sequencing, with results divided into two groups and sorted according to *p*‐values. (C) A heatmap illustrating the results of transcriptomic sequencing in C4‐2R cells, comparing low IGF1R expression with the negative control. (D) Gene set enrichment analysis (GSEA) showing that RNA expression of the oxidative phosphorylation pathway was upregulated when IGF1R expression was low. (E) GSEA indicating that RNA expression of the hypoxia pathway was upregulated under low IGF1R expression. (F) KEGG enrichment analysis of transcriptomic sequencing results, sorted according to *p* values. (G) A boxplot showing targeted metabolomics results for key metabolites. ‘Sc‐Vec’ represents the C4‐2R cell line as the negative control, ‘sh‐Vec’ represents the C4‐2R cell line with IGF1R knockdown, and ‘sh‐MICU1’ represents the cell line overexpressing MICU1 while knocking down IGF1R (*p < 0.05, **p < 0.01, ***p < 0.001). (H) The NAD+/NADH ratio from targeted metabolomics sequencing (*p < 0.05, **p < 0.01). (I) Schematic illustrating the proposed mechanism. IGF1R promotes ROS production by facilitating the influx of mitochondrial Ca^2+^, thereby trapping electrons in the electron transport chain. The dotted region indicates mitochondrial damage, impairing normal cellular processes.

### 3.3 IGF1R Is Associated With the MICU1

To further investigate the relationship between IGF1R and mitochondria, we explored the association between IGF1R and MICU1, a protein closely linked to mitochondrial Ca^2+^ regulation. RNA sequencing results were validated by qPCR in C4‐2R cells (Figure 3A). Western blot analysis confirmed a reduction in calcium channel‐related protein expression following IGF1R knockdown (Figure 3B). GeneCards database analysis suggested that CREB1, a transcription factor located on the PI3K pathway, may regulate MICU1 expression [10]. Notably, IGF1R knockdown led to decreased P‐CREB1 levels, which in turn reduced MICU1 expression in both C4‐2R (Figure 3C) and LNR (Figure S3A) cell lines. A rescue experiment further validated this observation in both C4‐2R (Figure 3D) and LNR (Figure S3B) cells. To explore the mechanism by which IGF1R regulates MICU1 through P‐CREB1, we used AKTi, an AKT phosphorylation inhibitor, to examine the effect of IGF1R on P‐CREB1 and MICU1 regulation (Figure 3E). ChIP experiments confirmed that P‐CREB1 binds to the MICU1 promoter region as a transcription factor in C4‐2R cells (Figure 3F). Using the UCSC Genome Browser [16], we retrieved the MICU1 promoter region and constructed dual‐luciferase reporter plasmids. The dual‐luciferase assay demonstrated that P‐CREB1 enhances MICU1 transcription and identified potential binding sites (Figure 3G). Another dual‐luciferase assay, performed with a plasmid containing mutated binding sites, confirmed the binding of P‐CREB1 to MICU1 (Figure 3H,I).

**FIGURE 3:**
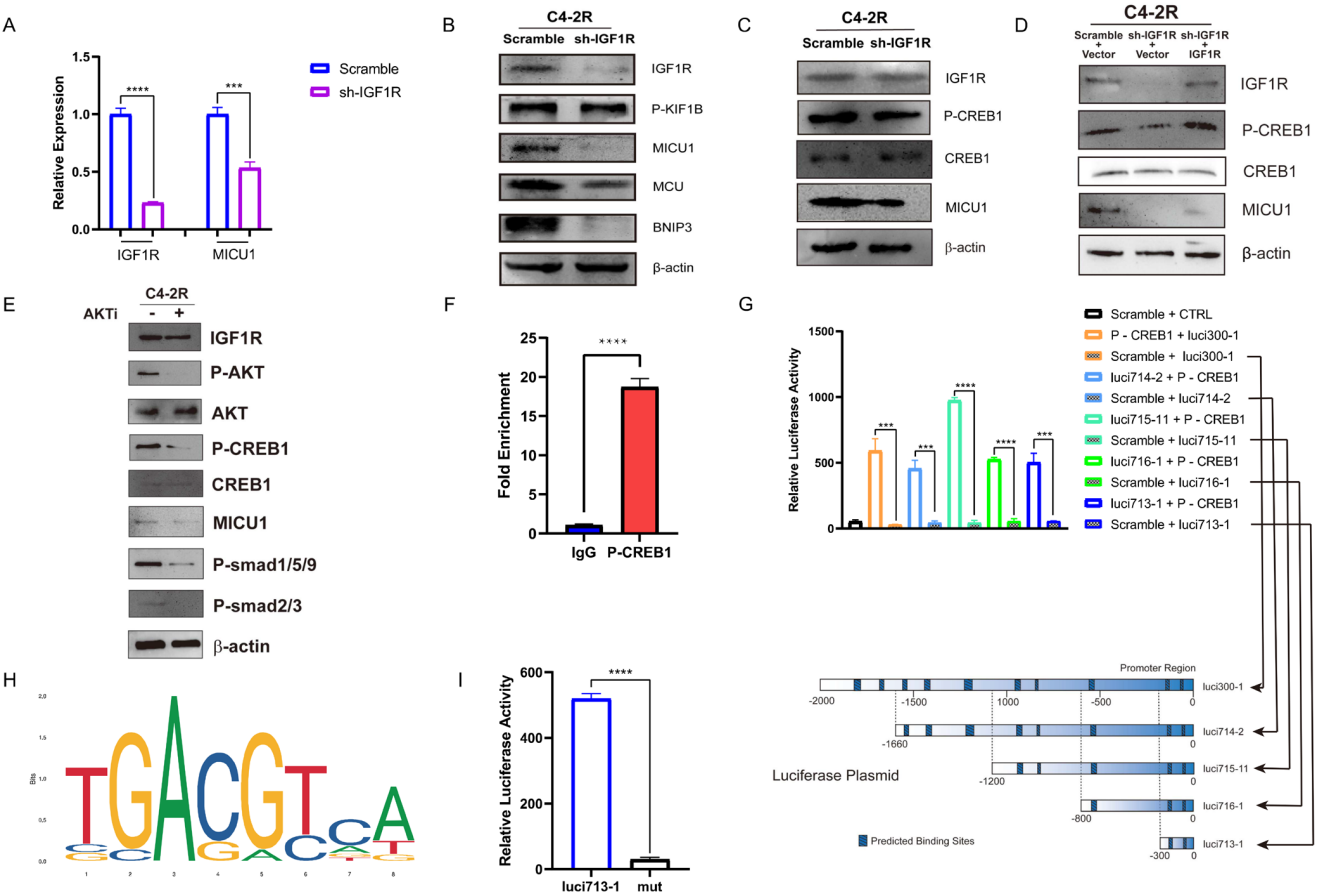
IGF1R regulates the expression of MICU1 through P‐CREB1. (A) The qPCR results indicated that knocking down IGF1R significantly affected MICU1 expression at the transcriptional level (***p < 0.001; ****p < 0.0001). (B) The image demonstrates a significant downregulation of MICU1‐related protein expression following IGF1R knockdown, alongside alterations in the expression of mitochondrial Ca^2+^‐related proteins. (C) Following IGF1R knockdown, there was a notable decrease in CREB1 phosphorylation, which was accompanied by reduced MICU1 expression. (D) Rescue experiments show that after IGF1R knockdown, the expression of P‐CREB1 and MICU1 varies in response to changes in IGF1R expression. (E) The AKT phosphorylation inhibitor, AKTI, was used to treat C4‐2R cells to observe changes in downstream expression. The concentration of AKTI was 2.5 μM. (F) ChIP assay results confirmed that P‐CREB1 binds to the MICU1 promoter as a transcription factor. (G) Upper panel: Dual‐luciferase assay results show that P‐CREB1 enhances MICU1 transcription. Lower panel: Diagram of the dual‐luciferase plasmid, truncated for experimental purposes. The arrow indicates the relative plasmid, and the numbers represent the base pairs from the MICU1 gene (***p < 0.001; ****p < 0.0001). (H) Schematic representation of the JASPAR database prediction for P‐CREB1 and MICU1 binding sites. Due to the proximity of the two predicted binding sites in the final plasmid, the results are shown as a single binding site. (I) Fluorescence data from the dual‐luciferase assay following mutation of the predicted binding site (****p < 0.0001).

### 3.4 IGF1R Plays a Central Role in Enzalutamide Resistance via MICU1

We first examined changes in mitochondrial Ca^2+^ distribution. To do this, we labelled all intracellular calcium using Fluo‐4 and mitochondrial membrane‐associated calcium with Rhod‐3. This analysis revealed that knockdown of IGF1R altered Ca^2+^ distribution, which was consistent with changes in MICU1 expression (Figure 4A). Fluorescent intensity measurements were performed using ImageJ software (Figure 4B,C). Additionally, we observed a decrease in mitochondrial Ca^2+^ levels, which was accompanied by a reduction in ROS, as confirmed by ROS fluorescence imaging (Figure 4D). Further fluorescent intensity measurements supported this observation (Figure 4E). We also investigated the TGF‐β signalling pathway, known to be regulated by ROS, as indicated in Figure 2. Western blot analysis revealed significant changes in SMAD family phosphorylation, demonstrating alterations in the TGF‐β pathway in both C4‐2R and LNR cells (Figure 4F, Figure S3C). Phosphorylation of SMAD1/5 and SMAD2/3 decreased with IGF1R knockdown. In contrast, IGF1R overexpression in C4‐2 cells led to increased MICU1 expression and elevated phosphorylation of the SMAD family (Figure 4G, Figure S3D). To strengthen the evidence, we knocked down MICU1 in C4‐2R cells and assessed the corresponding downstream protein changes (Figure 4H). Furthermore, IGF1R knockdown significantly reduced the expression of epithelial‐mesenchymal transition (EMT)‐related markers in C4‐2R and LNR cells (Figure 4I, Figure S3E), whereas IGF1R overexpression in C4‐2 cells enhanced EMT marker expression (Figure 4J). To further validate these findings, we overexpressed MICU1 in C4‐2R cells, resulting in corresponding changes in downstream proteins (Figure 4K). Finally, FISH experiments were conducted to assess the expression of three typical EMT proteins in C4‐2R cells, confirming the consistency of these results with our previous observations (Figure 4L).

**FIGURE 4:**
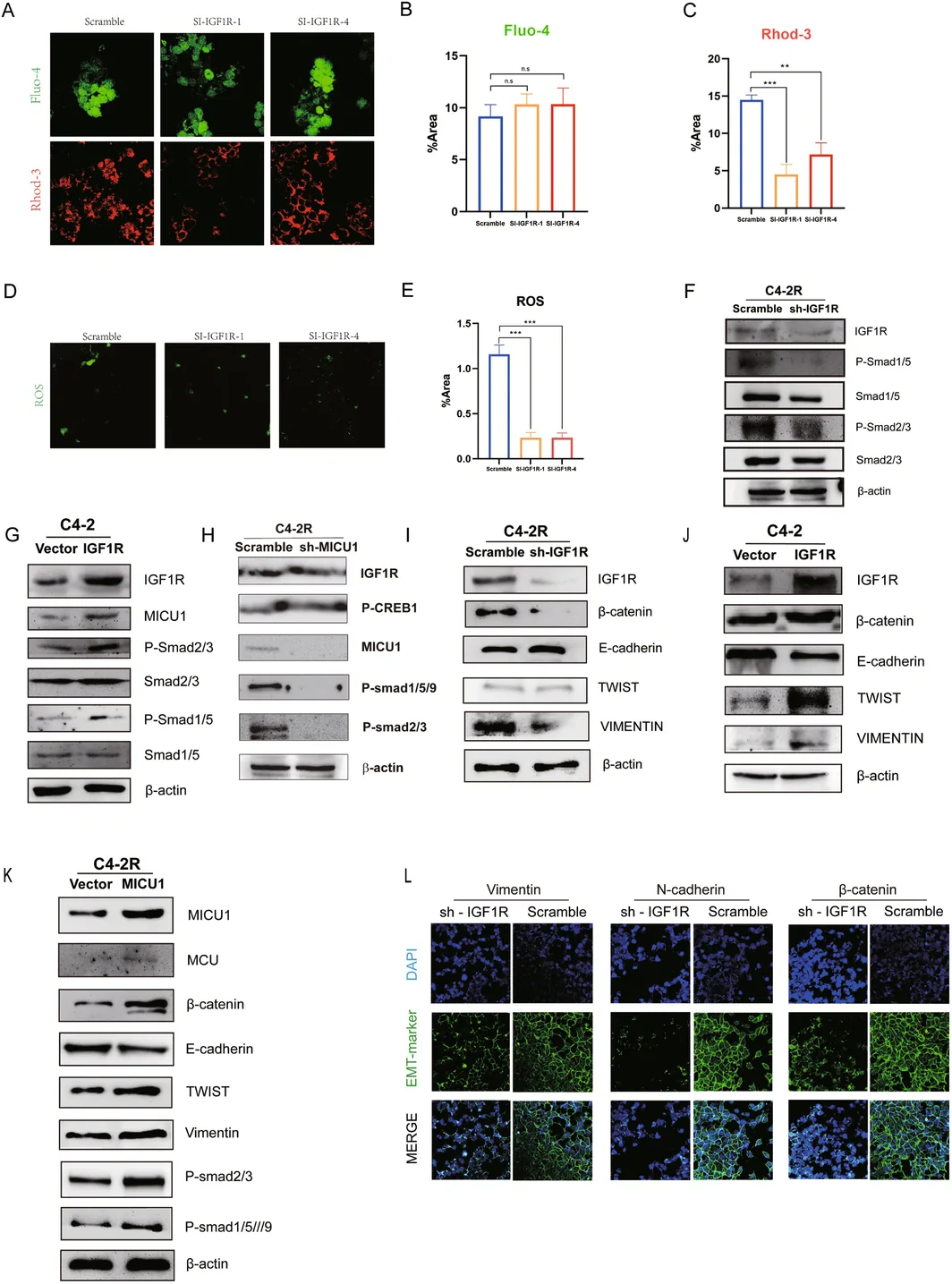
IGF1R can change the concentration of mitochondrial Ca^2+^ by MICU1, which in turn affects the production of ROS. (A) The distribution of Ca^2+^ in cells was assessed using two different fluorescence markers. Fluo‐4 indicates the total Ca^2+^ levels, while Rhod‐3 specifically detects Ca^2+^ located in internal membranes. After IGF1R knockdown, the total Ca^2+^ levels remained unchanged, but the Ca^2+^ associated with intracellular membranes was significantly reduced (Rhod‐3 labels and colours only Ca^2+^ located in the cell membrane). (B) Statistical analysis of Fluo‐4 fluorescence intensity, calculated using ImageJ software (**p < 0.01, ***p < 0.001). (C) Statistical analysis of Rhod‐3 fluorescence intensity, calculated using ImageJ software (**p < 0.01, ***p < 0.001). (D) Low IGF1R expression resulted in decreased ROS production. (E) Statistical analysis of ROS fluorescence intensity, calculated using ImageJ software (***p < 0.001). (F) SMAD family phosphorylation levels vary with IGF1R expression levels. (G) Overexpression of IGF1R in C4‐2 cell lines led to increased phosphorylation levels of the SMAD family. (H) Knockdown of MICU1 in C4‐2R cell lines resulted in decreased phosphorylation levels of SMAD family proteins. (I) Expression of EMT‐related markers following IGF1R knockdown in C4‐2R cell lines. (J) Expression of EMT‐related markers following IGF1R overexpression in C4‐2 cell lines. (K) Overexpression of MICU1 in C4‐2R cells resulted in altered downstream protein expression, with trends consistent with changes in IGF1R expression. (L) Fluorescence changes in EMT‐related markers after reduced IGF1R expression.

### 3.5 Knocking Down IGF1R Changes Several Phenotypes of Enzalutamide‐Resistance Cell Lines

We analyzed the alterations in cell phenotype following IGF1R knockdown. Knockdown of IGF1R significantly reduced both metastatic (Figure 5A,B) and proliferative abilities (Figure 5C,D) in all enzalutamide‐resistant cell lines. In contrast, overexpression of MICU1 in C4‐2R cells with IGF1R knockdown effectively reversed these cellular phenotypic changes and restored sensitivity to enzalutamide (Figure 5E–G).

**FIGURE 5:**
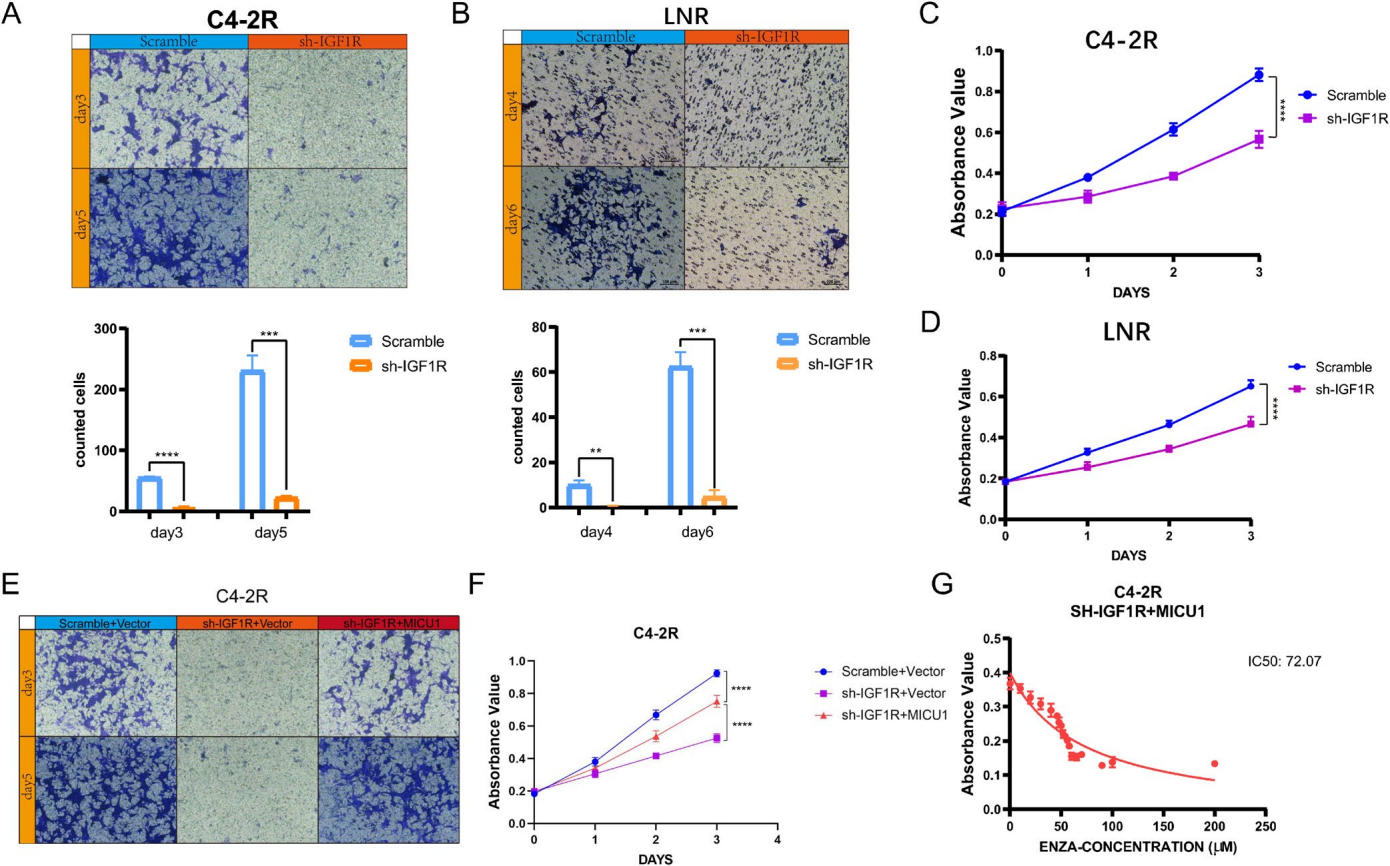
Knocking down IGF1R can lead to many phenotypic changes in cells. (A) Transwell migration assay results after IGF1R knockdown in C4‐2R cell lines (**p < 0.01, ***p < 0.001). (B) Transwell migration assay results after IGF1R knockdown in LNR cell lines (***p < 0.001, ****p < 0.0001). (C) MTT assay results after IGF1R knockdown in C4‐2R cell lines (**p < 0.01, ***p < 0.001). (D) MTT assay results after IGF1R knockdown in LNR cell lines (**p < 0.01, ***p < 0.001). (E) Overexpression of MICU1 in IGF1R‐knockdown C4‐2R cell lines restores migratory capacity, which was impaired by low IGF1R levels. (F) Overexpression of MICU1 in IGF1R‐knockdown C4‐2R cell lines restores proliferative capacity, which was impaired by low IGF1R levels (**p < 0.01, ***p < 0.001). (G) Overexpression of MICU1 restores C4‐2R sensitivity to enzalutamide, which was reduced by IGF1R knockdown.

### 3.6 IGF1R Has the Ability to Be Used as a Therapeutic Target

To investigate the relationship between IGF1R and cellular sensitivity to enzalutamide, we conducted a series of experiments. First, we used Linsitinib, an IGF1R inhibitor, to assess its effect on cell growth in various culture conditions, optimizing the working concentration to 50 μM (Figure S3F). The results showed that Linsitinib enhanced the effects of enzalutamide, whereas IGF1R overexpression increased cellular resistance to enzalutamide (Figure 6A,B). Additionally, in vitro assays were performed to evaluate the potential of IGF1R as a therapeutic target. Linsitinib was used to inhibit IGF1R phosphorylation in enzalutamide‐resistant cell lines (Figure 6C,E). The combination of enzalutamide and Linsitinib significantly reduced cell viability in these cell lines (Figure 6D,F). We also examined the role of IGF1R in tumour tissue using a xenograft model, where mice were divided into a treatment group (Linsitinib and enzalutamide) and a control group (enzalutamide alone) (Figure 6G). Tumours in the treatment group exhibited slower growth and smaller final size compared to the control group (Figure 6H,I). Immunohistochemical analysis showed significant inhibition of IGF1R phosphorylation and downstream proteins in the treatment group (Figure 6J). We also compared the tumour growth rates in the other two treatment methods: negative control group (only treating with DMSO) and Linsitinib treating solely group (Figure S4A,B).

**FIGURE 6:**
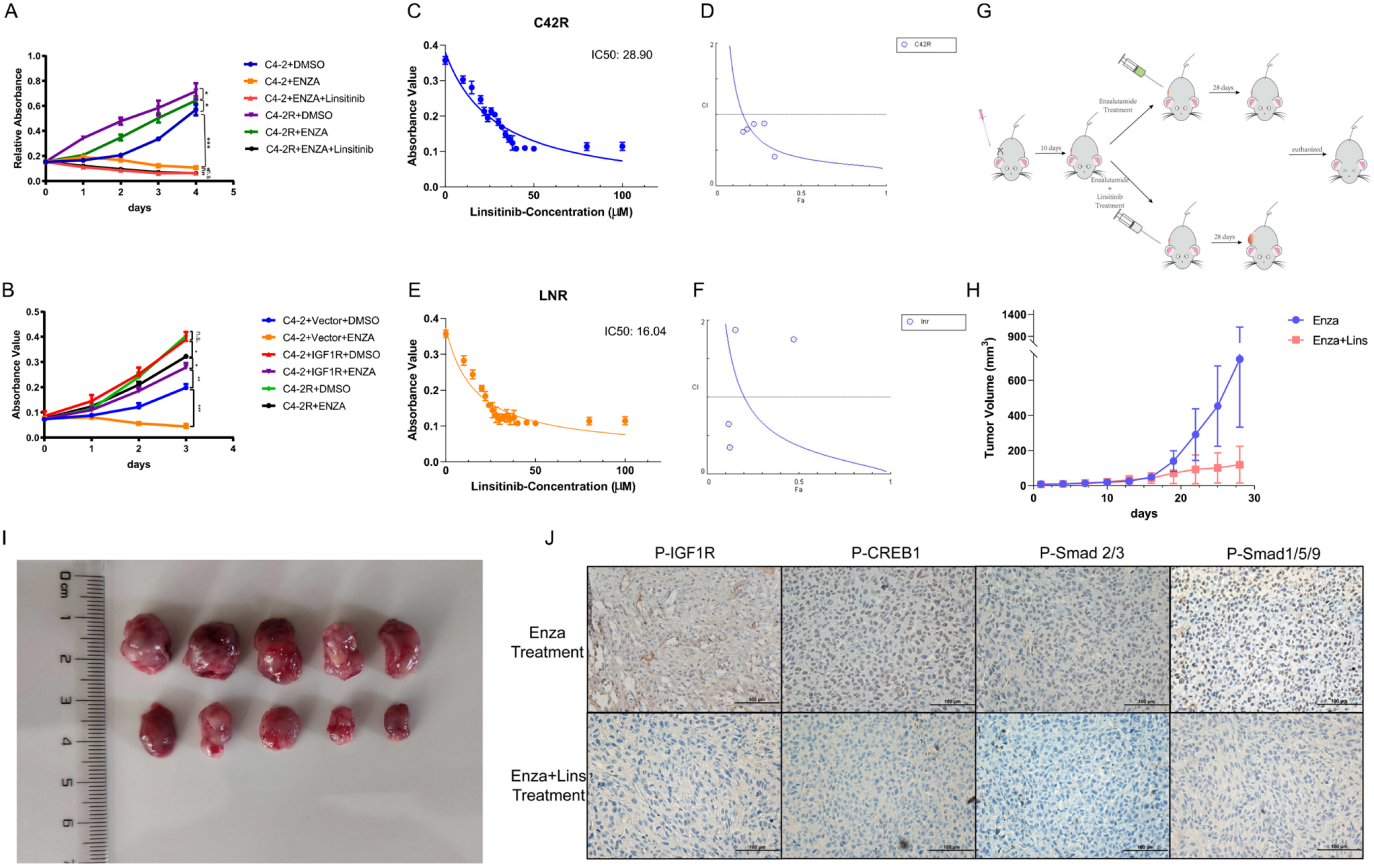
IGF1R has great potential as a therapeutic target. (A) Inhibition of IGF1R with Linsitinib increases the sensitivity of C4‐2R cells to enzalutamide. All cell lines were cultured in RPMI 1640 medium, with enzalutamide maintained at a concentration of 20 μM, the standard concentration for C4‐2R cells in routine culture. Linsitinib was used at a concentration of 50 μM (*p < 0.05, **p < 0.01, ***p < 0.001). (B) Overexpression of IGF1R in C4‐2 cells leads to increased resistance to enzalutamide. The enzalutamide concentration was maintained at 20 μM, the standard concentration for C4‐2 cells in routine culture (*p < 0.05, **p < 0.01, ***p < 0.001). (C) IC50 values for C4‐2R. The X‐axis represents the concentration of Linsitinib. (D) The drug combination curve illustrates the effect of the Linsitinib‐enzalutamide combination on C4‐2R cell lines. (E) IC50 values for LNR. The X‐axis represents the concentration of Linsitinib. (F) The drug combination curve illustrates the effect of the Linsitinib‐enzalutamide combination on LNR cell lines. (G) Schematic diagram of animal experiments. (H) Statistical analysis of tumour volume during animal experiments. Tumour volume was calculated using the formula described in the Methods section. In the figure, ‘Enza’ refers to enzalutamide, while ‘Enza + Lins’ denotes the combination treatment of enzalutamide and Linsitinib. (I) Tumour tissues were harvested following euthanasia of mice, and their volumes were compared under identical conditions. (J) IHC results showing the expression of key proteins in tumour tissue.

## 4 Discussion

Over time, cancer has emerged as a major threat to human health, with prostate cancer being a leading cause of mortality among adult men [2]. Advanced prostate cancer is typically treated with ADT, which has been shown to improve progression‐free survival in patients [17]. Despite its efficacy, there is no cure for advanced prostate cancer, and drug resistance remains a significant challenge after ADT treatment [18]. Enzalutamide, a commonly used drug in post‐ADT treatment, has been widely adopted, but enzalutamide resistance in patients has become a growing clinical concern. Therefore, identifying a therapeutic target that can be effectively used in clinical settings is critical.

To address this, we utilized an enzalutamide‐resistant cell line as a model to further investigate the mechanisms of drug resistance and identify potential therapeutic targets. Proteomic sequencing revealed that IGF1R, a key gene not yet thoroughly studied in the context of enzalutamide resistance, was significantly upregulated in C4‐2R cell lines. IGF1R, a classical upstream regulator of the PI3K pathway, is widely used in clinical treatments [19]. Therefore, we aimed to explore the specific role of IGF1R in enzalutamide resistance.

Our phosphorylation proteomics indicated that knocking down IGF1R altered the TGF‐β pathway. Subsequent transcriptomic analysis suggested that this alteration might be mediated by ROS [6]. IGF1R knockdown led to increased levels of TCA cycle intermediates and the expression of oxidative phosphorylation‐related proteins, while the NAD^+^/NADH ratio decreased. Analysing prior sequencing data, we also found that IGF1R significantly affected the calcium signalling pathway. Mitochondrial Ca^2+^ concentration, which directly influences oxidative phosphorylation and ROS production, is implicated in the complex mechanisms underlying drug resistance [20]. We hypothesize that IGF1R promotes ROS production by modulating mitochondrial Ca^2+^ levels, particularly when the inner mitochondrial membrane (IMM) is damaged, as observed in drug‐resistant cell lines. The abnormal phenomena observed at low IGF1R levels appear to be compensatory mechanisms. To further investigate, we overexpressed MICU1, a protein directly involved in mitochondrial Ca^2+^ regulation, following IGF1R knockdown, and assessed the recovery of specific metabolites through targeted metabolomics. Preliminary sequencing results supported our hypothesis.

Next, we examined the impact of IGF1R on MICU1 expression. We found that reduced IGF1R expression led to a decrease in MICU1 at the transcriptional level. This prompted us to explore whether transcription factors regulated by IGF1R were involved in MICU1 expression. Database analyses (JASPAR [11], GENECARDS [10]) suggested that P‐CREB1 could bind to the MICU1 promoter region. P‐CREB1, a downstream mediator of IGF1R, functions as a transcription factor upon phosphorylation. Dual‐luciferase assays confirmed the binding of P‐CREB1 to the MICU1 promoter. We also examined the downstream effects on MCU and observed significant links to changes in ROS and Ca^2+^ distribution. Our hypothesis aligns with the reported role of the TGF‐β pathway [21] in CRPC and its association with ROS modulation. Furthermore, EMT is known to contribute to cell resistance [22], and we validated the expression of the SMAD family to support our hypothesis.

To further test our hypothesis, we conducted transwell assays to evaluate changes in cell phenotype and MTT assays to assess cell proliferation. We also measured the IC50 values of the corresponding cell lines to investigate the relationship between IGF1R and enzalutamide resistance. Our results indicated that IGF1R knockdown significantly inhibited cell proliferation and migration. Additionally, treatment with Linsitinib, a phosphorylation inhibitor of IGF1R, demonstrated that higher levels of IGF1R expression are associated with increased resistance to enzalutamide [23]. At a concentration of 50 μM, Linsitinib inhibited the growth of both C4‐2R and C4‐2 cell lines in 1640 medium containing 20 μM enzalutamide. Overexpression of IGF1R in C4‐2 cells also increased resistance to enzalutamide. To assess the therapeutic potential of IGF1R in enzalutamide‐resistant cell lines, we conducted both in vitro and in vivo experiments. In vitro, we constructed dose–response curves for C4‐2R and LNR cells treated with a combination of enzalutamide and Linsitinib. In vivo, we performed a xenograft assay to evaluate the effects of IGF1R‐targeted therapy on tumour growth, with protein expression in tumour tissue analyzed by IHC.

This study reveals that IGF1R can influence ROS production by altering mitochondrial Ca^2+^ distribution (Figure 7). Two major innovations in this work are: first, the discovery of a novel metabolic mechanism of IGF1R in post‐ADT treatment cell lines, and second, the identification of a metabolic link between two cell signalling pathways. Given that IGF1R is already a widely utilized therapeutic target in clinical practice, our findings underscore its potential as a novel therapeutic target for prostate cancer treatment. This research successfully integrates metabolomics and proteomics, offering a new approach to studying and regulating cellular signalling pathways. Although ROS play a pivotal role in cellular resistance, research on this topic in prostate cancer is often overlooked due to the predominant focus on androgen receptor (AR) signalling. However, the role of AR is not fully understood in prostate cancer patients post‐ADT or in those with congenital drug resistance [24]. Our study highlights the importance of exploring non‐AR‐related pathways in prostate cancer.

**FIGURE 7:**
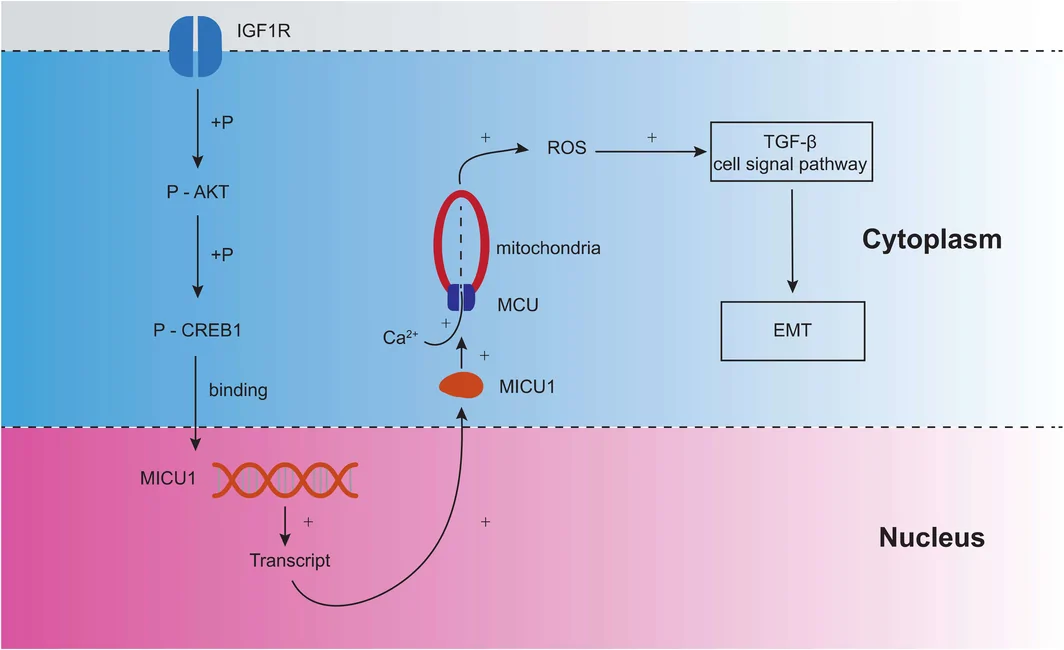
Mechanism schematic diagram.

## Author Contributions

**Hanlin Li:** conceptualization, methodology, validation, investigation, visualization, writing – review and editing, writing – original draft. **Yutong Chen:** methodology. **Yupeng Zhou:** methodology. **Yang Li:** methodology. **Yukui Gao:** methodology. **Xuanrong Chen:** supervision, data curation. **Qianwang Ma:** software, formal analysis, project administration. **Shimiao Zhu:** funding acquisition. **Simeng Wen:** visualization, project administration.

## Funding

This work was supported by the National Natural Science Foundation of China (No. 82272918, 82072851, 82061160493 to Z.S. 81972654, 82273262 to S.W, 82172759 to S.Z and No. 82172829 to Y.N.).

## Ethics Statement

Approval of the research protocol by an Institutional Reviewer Board. Animal Studies: All animal experiments have obtained animal ethics approval from The Second Affiliated Hospital, Zhejiang University School of Medicine.

## Consent

The authors have nothing to report.

## Conflicts of Interest

The authors declare no conflicts of interest.

## Supporting information


**Figure S1:** (A) The picture shows the IC50 results of the LNCaP cell line. The X‐axis represents the different enzalutamide concentrations, and the Y‐axis represents the absorbance of C4‐2 cells in MTT assay at the corresponding enzalutamide concentration. (B) The picture shows the IC50 results of the C4‐2R cell line. The X‐axis represents the different enzalutamide concentrations, and the Y‐axis represents the absorbance of C4‐2R cells in MTT assay at the corresponding enzalutamide concentration. To contrast with I, the C4‐2R cell line was transfected with a negative control plasmid. (C) The picture shows the IC50 results of the C4‐2R cell line with low IGF1R expression. The X‐axis represents the different enzalutamide concentrations, and the Y‐axis represents the absorbance of sh‐IGF1R‐C4‐2R cells in MTT assay at the corresponding enzalutamide concentration.

**Figure S2:** (A) A volcano blot based on the results of the anionic portion of the metabolomics sequencing results, between C4‐2 and C4‐2R. (B) A volcano blot based on the results of the cationic portion of the metabolomics sequencing results. (C) Heatmap based on the results of the anionic portion of the metabolomics sequencing results. (D) Heatmap based on the results of the cationic portion of the metabolomics sequencing results. (E) KEGG enrichment results of the metabolomics sequencing. (F) The boxplot results demonstrate targeted metabolomics results for some of the important products (*p < 0.05, **p < 0.01, ***p < 0.001).

**Figure S3:** (A) After knocking down IGF1R, the phosphorylation level of CREB1 decreased, and the expression of MICU1 also decreased. (B) Rescue experiments show that after knocking down IGF1R, the expression of P‐CREB1 and MICU1 changes with the change of IGF1R expression. (C) SMAD family phosphorylation levels vary with the expression level of IGF1R. (D) Overexpression of IGF1R in LNCaP cell lines increased phosphorylation levels in the smad family. (E) Expression of EMT‐related markers after knocking down IGF1R in C4‐2R cell lines. (F) Results of Linsitinib's inhibition of C4‐2R cell lines. Cells were cultured in enzalutamide‐free 1640 medium. The X axis in the figure is the number of days cells are cultured in 1640 medium containing Linsitinib.

**Figure S4:** (A) Xenograft module contains other two treatment methods: negative control group (only treating with DMSO) and Linsitinib treating soley group. In the figure, ‘Enza’ means enzalutamide, while ‘Enza+Lins’ means the combination treatment of enzalutamide and Linsitinib. (B) Statistical results of tumour volume during animal experiments. Tumour volume is calculated based on the formula listed in the Methods section.

**Table S1:** Sequence of si‐IGF1R.
**Table S2:** Antibodies and catalogues used in this project.
**Table S3:** Concentration of drugs used in the fa‐ci assay.

## Data Availability

The data that support the findings of this study are available from the corresponding author upon reasonable request.
