# Supplementary material for: IGF1R Induces ROS Formation in Mitochondria via MICU1 Leads to the Enzalutamide Resistance in Prostate Cancer
Source: J Cell Mol Med. 2026 Oct 27;30(20):e71415. doi: 10.1111/jcmm.71415 (PMC13711022; doi:10.1111/jcmm.71415)
Supplement: Supplementary file 1 [file JCMM-30-e71415-s001.pdf]

Figure. S4

A

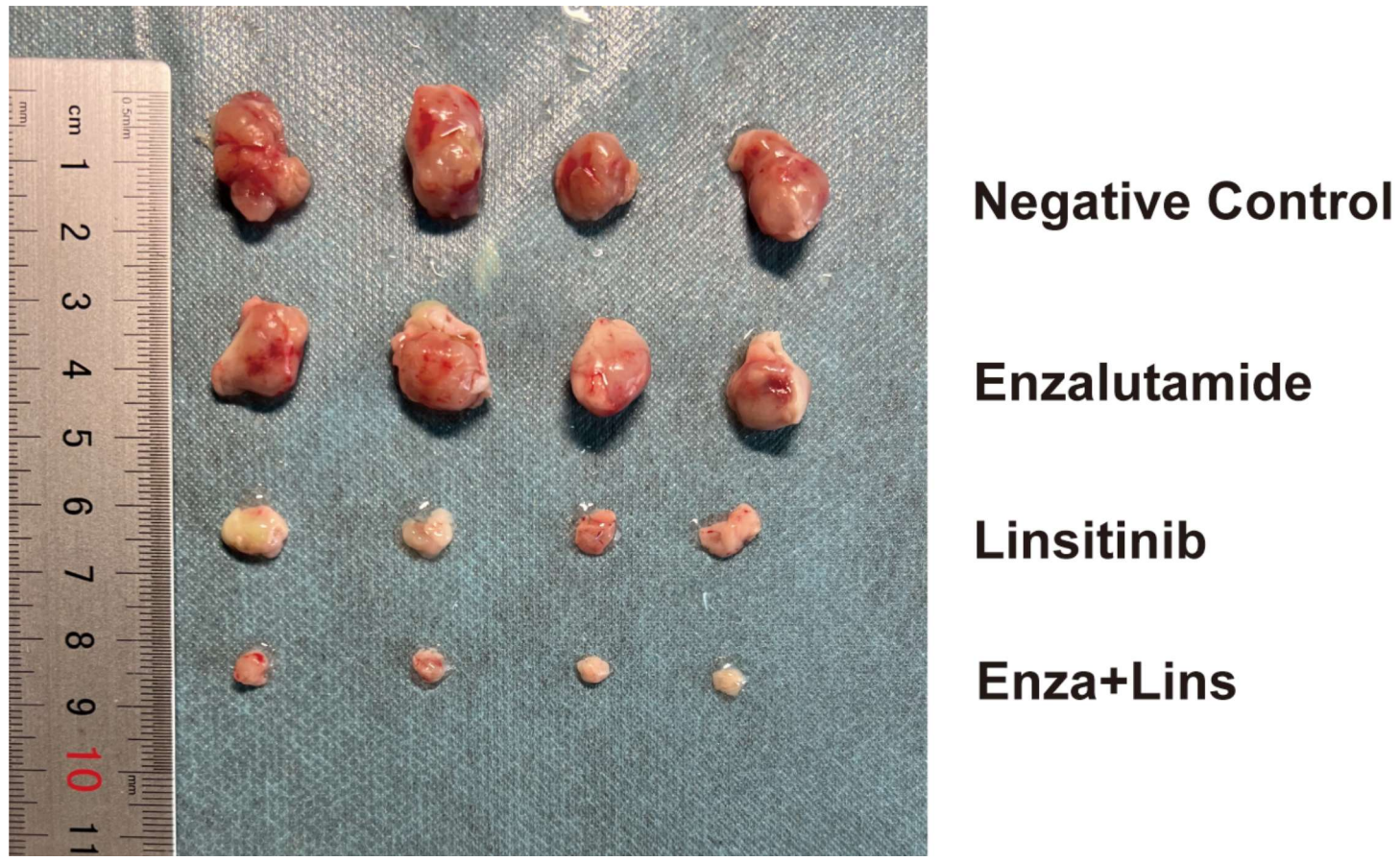
Negative Control
Enzalutamide
Linsitinib
Enza+Lins

B

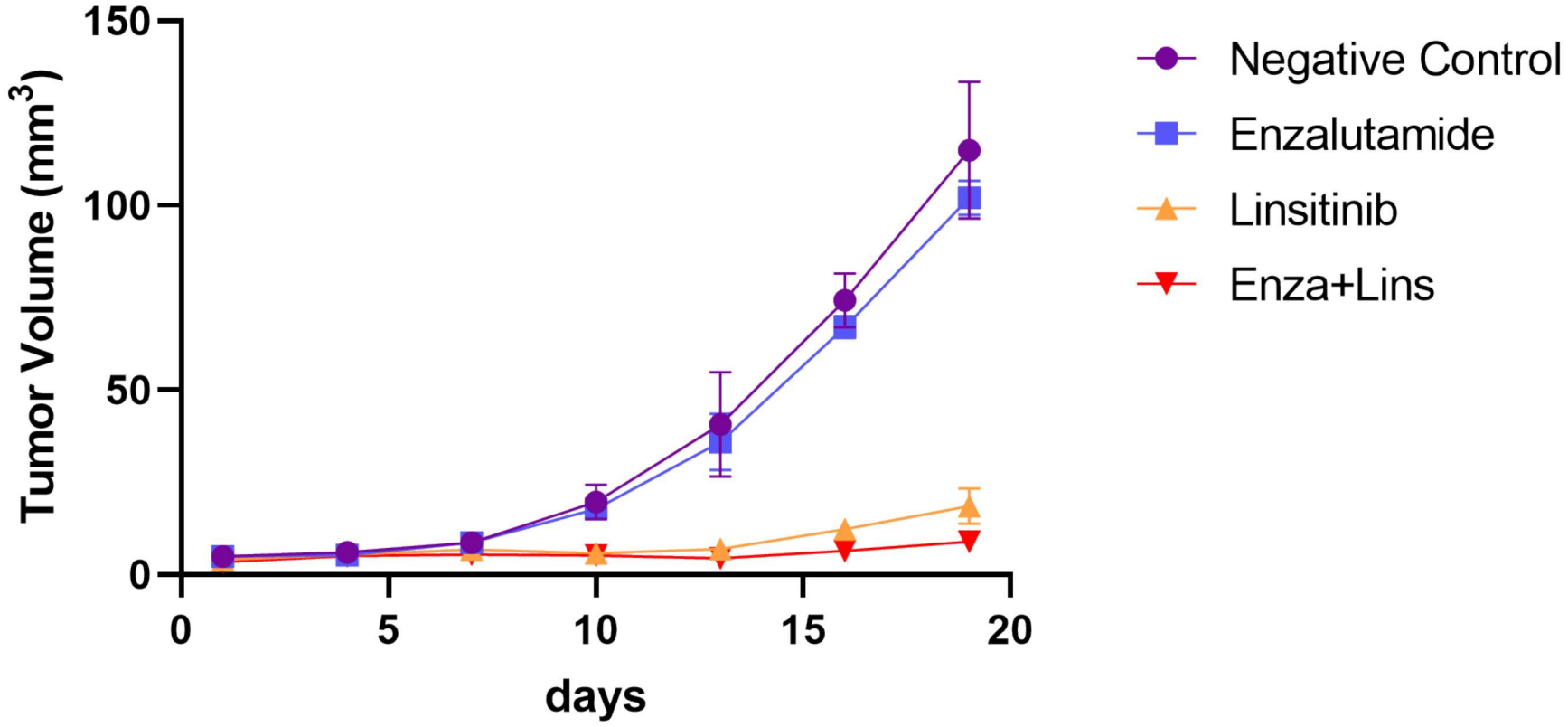
150
100
50
0
Tumor Volume (mm3)
0
5
10
15
20
days
Negative Control
Enzalutamide
Linsitinib
Enza+Lins
