# Supplementary material for: IGF1R Induces ROS Formation in Mitochondria via MICU1 Leads to the Enzalutamide Resistance in Prostate Cancer
Source: J Cell Mol Med. 2026 Oct 27;30(20):e71415. doi: 10.1111/jcmm.71415 (PMC13711022; doi:10.1111/jcmm.71415)
Supplement: Supplementary file 2 [file JCMM-30-e71415-s002.pdf]

Figure. S1

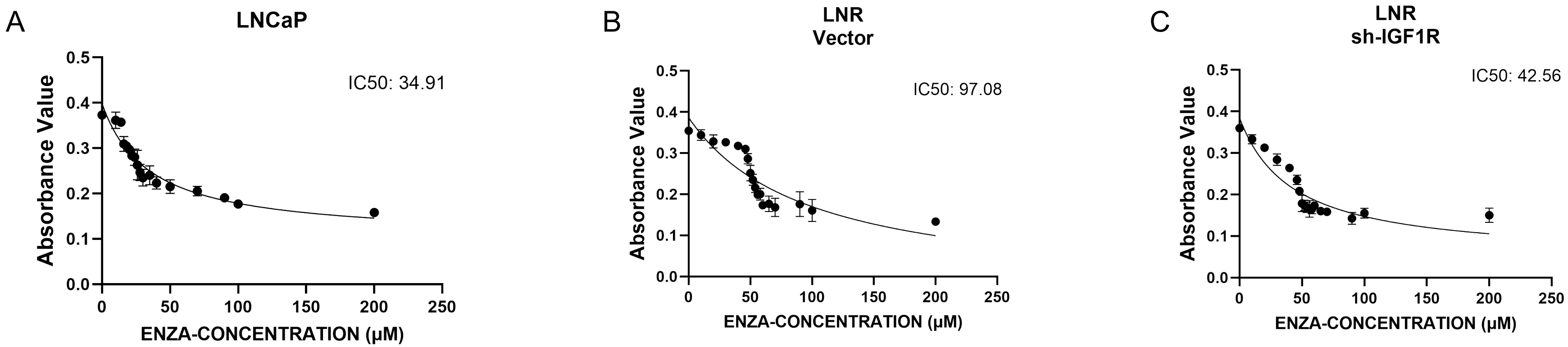

A
LNCaP
IC50: 34.91
Absorbance Value
ENZA-CONCENTRATION (μM)
B
LNR
Vector
IC50: 97.08
Absorbance Value
ENZA-CONCENTRATION (μM)
C
LNR
sh-IGF1R
IC50: 42.56
Absorbance Value
ENZA-CONCENTRATION (μM)
