# Supplementary material for: IGF1R Induces ROS Formation in Mitochondria via MICU1 Leads to the Enzalutamide Resistance in Prostate Cancer
Source: J Cell Mol Med. 2026 Oct 27;30(20):e71415. doi: 10.1111/jcmm.71415 (PMC13711022; doi:10.1111/jcmm.71415)
Supplement: Supplementary file 3 [file JCMM-30-e71415-s003.pdf]

Figure. S3

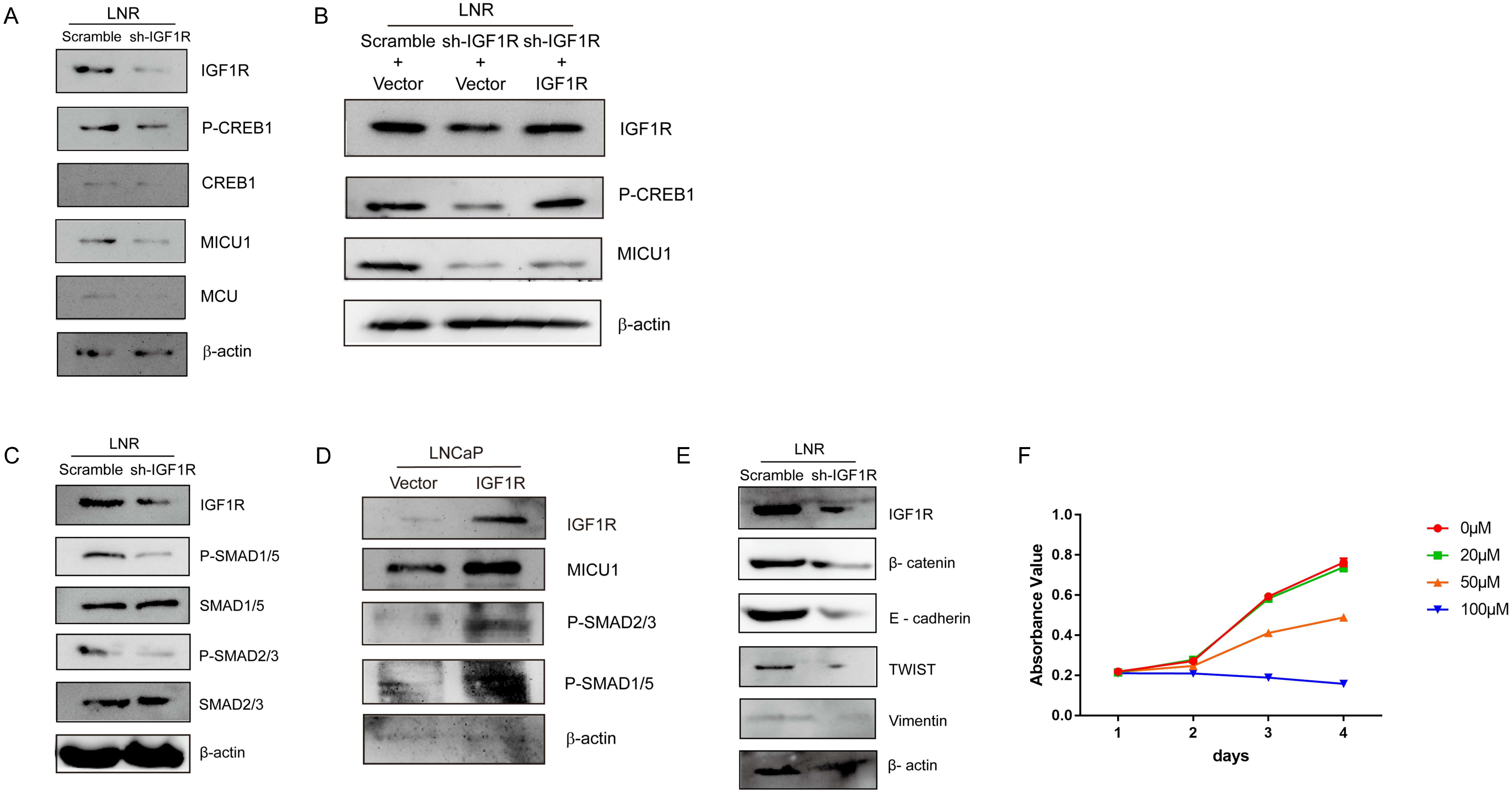
A
LNR
Scramble sh-IGF1R
IGF1R
P-CREB1
CREB1
MICU1
MCU
β-actin
B
LNR
Scramble + Vector
sh-IGF1R + Vector
sh-IGF1R + IGF1R
IGF1R
P-CREB1
MICU1
β-actin
C
LNR
Scramble sh-IGF1R
IGF1R
P-SMAD1/5
SMAD1/5
P-SMAD2/3
SMAD2/3
β-actin
D
LNCaP
Vector IGF1R
IGF1R
MICU1
P-SMAD2/3
P-SMAD1/5
β-actin
E
LNR
Scramble sh-IGF1R
IGF1R
β- catenin
E - cadherin
TWIST
Vimentin
β- actin
F
Absorbance Value
1.0
0.8
0.6
0.4
0.2
0.0
1
2
3
4
days
0μM
20μM
50μM
100μM
