# Supplementary material for: IGF1R Induces ROS Formation in Mitochondria via MICU1 Leads to the Enzalutamide Resistance in Prostate Cancer
Source: J Cell Mol Med. 2026 Oct 27;30(20):e71415. doi: 10.1111/jcmm.71415 (PMC13711022; doi:10.1111/jcmm.71415)
Supplement: Supplementary file 4 [file JCMM-30-e71415-s004.pdf]

Figure. S2

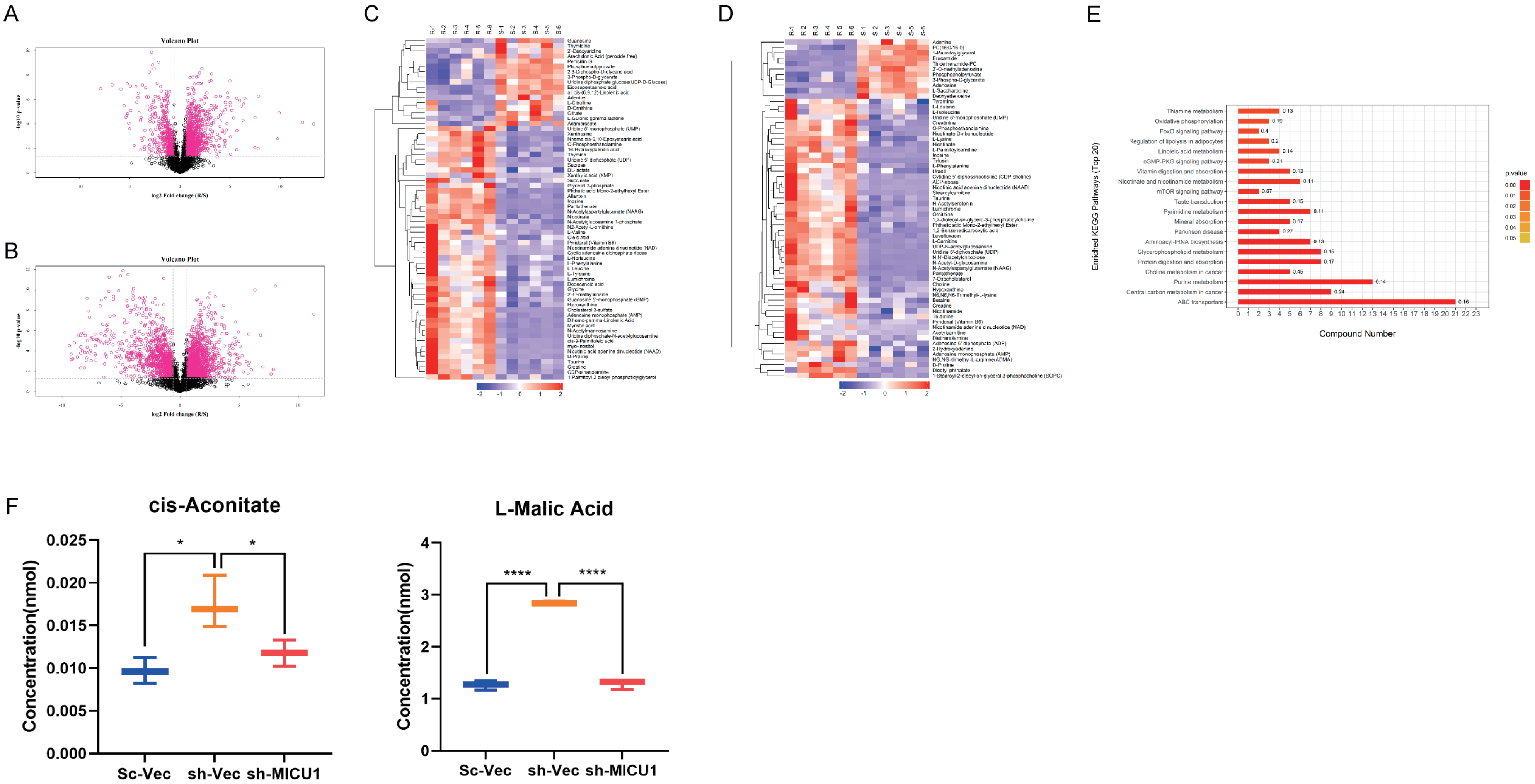

A
Volcano Plot
log2 Fold change (R/S)
B
Volcano Plot
log2 Fold change (R/S)
C
D
E
Enriched KEGG Pathways (Top 20)
Compound Number
p.value
F
cis-Aconitate
Concentration(nmol)
Sc-Vec
sh-Vec
sh-MICU1
L-Malic Acid
Concentration(nmol)
Sc-Vec
sh-Vec
sh-MICU1
