# Supplementary material for: IGF1R Induces ROS Formation in Mitochondria via MICU1 Leads to the Enzalutamide Resistance in Prostate Cancer
Source: J Cell Mol Med. 2026 Oct 27;30(20):e71415. doi: 10.1111/jcmm.71415 (PMC13711022; doi:10.1111/jcmm.71415)
Supplement: Supplementary file 5 [file JCMM-30-e71415-s005.docx]

Supplementary Table 1

| SiIGF1R-1 | CCACCAUCAACAAUGAGUATT  UACUCAUUGUUGAUGGUGGTT |
| --- | --- |
| Si-IGF1R-2 | GGAGUCUUCACCACUUACUTT  AGUAAGUGGUGAAGACUCCTT |
| Sh-MICU1 | GCUGGAGCAUCUCUUGAUATT  UAUCAAGAGAUGCUCCAGCTT |

Supplementary Table 2

| Antibody | Company | Product code |
| --- | --- | --- |
| IGF1R | abcam | ab182408 |
| P-IGF1R | Affinity | AF3125 |
| MICU1 | CST | 12524 |
| Smad2/3 | CST | 8685 |
| Phospho-Smad2/3 | CST | 8828 |
| Smad1 | CST | 6944 |
| Smad5 | abcam | ab40771 |
| Phospho-Smad1/5/9 | CST | 13820 |
| BNIP3 | abcam | ab109362 |
| MCU | CST | 14997 |
| Phospho-CREB1 | abcam | ab32096 |
| CREB1 | abcam | ab32515 |
| Phospho-KIF1B | CST | 11993 |
| BNIP3 | abcam | ab433 |
| β-actin | abcam | ab8227 |

Supplementary Table 3

| Cell Line | Concentration (μM) |
| --- | --- |
| C4-2R | (ENZA): 0, 10, 20, 30, 40, 46, 48, 50, 52, 54, 56, 58, 60, 65, 70, 90, 100, 200 |
| SHIGF1R-C4-2R | (ENZA): 0, 5, 10, 15, 20, 24, 28, 32, 34, 36, 38, 40, 42, 46, 50, 60, 80, 100, 150, 200 |
| C4-2 | (ENZA): 0, 10, 14, 16, 18, 20, 22, 24, 26, 28, 30, 35, 40, 50, 70, 90, 100, 200 |
| MICU1-C4-2 | (ENZA): 0, 10, 20, 30, 40, 46, 48, 50, 52, 54, 56, 58, 60, 65, 70, 90, 100, 200 |
| LNR | (ENZA): 0, 10, 20, 30, 40, 46, 48, 50, 52, 54, 56, 58, 60, 65, 70, 90, 100, 200 |
| SHIGF1R-LNR | (ENZA): 0, 10, 20, 30, 40, 46, 48, 50, 52, 54, 56, 58, 60, 65, 70, 90, 100, 200 |
| LNCaP | (ENZA): 0, 10, 14, 16, 18, 20, 22, 24, 26, 28, 30, 35, 40, 50, 70, 90, 100, 200 |
